# Iron metabolism dysregulation and post-stroke cognitive impairment: mechanisms and therapeutic perspectives

**DOI:** 10.3389/fneur.2026.1765621

**Published:** 2026-03-03

**Authors:** Zi-Ming Guo, Ying-Tong Lu, Jia-Qi Li, Hao-Qi Wu, Xi-Chen Zhu, Tao Ma

**Affiliations:** 1 Wuxi School of Medicine, Jiangnan University, Wuxi, Jiangsu, China; 2 Department of Neurology, The Wuxi No. 2 People’s Hospital, Jiangnan University Medical Center, Wuxi, Jiangsu, China; 3 Affiliated Wuxi Clinical College of Nantong University, Nantong, Jiangsu, China

**Keywords:** ferroptosis, iron chelating agent, iron deposition, iron metabolism, ketogenic diet, post-stroke cognitive impairment, traditional Chinese medicine

## Abstract

Post-stroke cognitive impairment (PSCI) is a frequent neurological consequence of acute stroke. It manifests as persistent deficits in cognitive function for at least 6 months following cerebral infarction and substantially diminishes patients’ quality of life. Currently, its specific pathogenesis remains unclear. In recent years, increasing attention has been directed toward the contribution of disrupted iron metabolism to the development of PSCI. Acute stroke can cause iron metabolism disorder in the central nervous system and result in iron deposition, which causes damage to nerve cells through mechanisms such as ferroptosis, thereby leading to cognitive decline. Therefore, studies on the treatment of PSCI by regulating this mechanism have emerged. This review summarizes recent advances in the mechanisms linking iron metabolism dysregulation to PSCI and highlights emerging therapeutic strategies, offering new insights for improving its diagnosis and management.

## Introduction

Post-stroke cognitive impairment (PSCI) is a prevalent complications of acute cerebral infarction, involving cognitive deficits that persist for at least 6 months after the initial event (1). PSCI is generally regarded as a clinical subtype within the broader spectrum of vascular cognitive impairment (VCI), specifically referring to cognitive deficits occurring after a clinically overt stroke event. Although PSCI markedly reduces patients’ quality of life, its underlying mechanisms remain incompletely understood, and epidemiological data indicate that more than one-third of stroke survivors are affected (2). In addition to impairing daily functioning, PSCI increases the risk of long-term disability and mortality. The dynamic balance of iron content maintains the normal operation of neurological function, and the occurrence of stroke can break this balance. Therefore, disturbances in iron metabolism are closely linked to stroke. Abnormal iron metabolism is mainly manifested as iron deposition in the brain, and may promote ferroptosis, which leads to nerve cell damage and dysfunction (3). Consequently, the interplay between iron metabolism disorders and PSCI has gained considerable research interest in recent years. This article first clarifies the physiological mechanisms of brain iron metabolism and the alterations that occur following stroke, then reviews the related research on iron deposition, ferroptosis and PSCI, and finally summarizes the regulatory effects of iron chelators, diet therapy and traditional Chinese medicine (TCM) on PSCI related to iron metabolism disorders, we propose that iron metabolism dysregulation promotes PSCI primarily through iron deposition-induced ferroptosis, and that targeting this axis represents a promising therapeutic strategy.

## Iron metabolism

**Figure 1:**
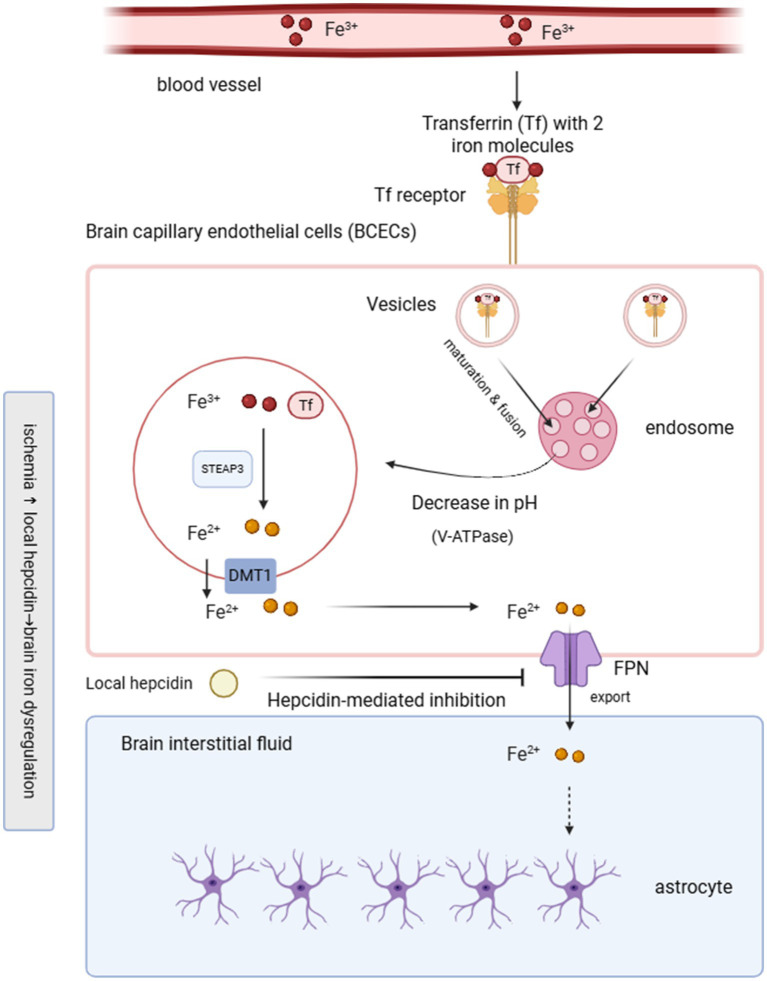
Iron transport across the blood–brain barrier and its dysregulation after ischemic stroke. Under physiological conditions, transferrin-bound Fe^3+^ (Tf–Fe^3+^) binds to transferrin receptor 1 (TfR1) on brain capillary endothelial cells (BCECs) and undergoes clathrin-mediated endocytosis. Within acidified endosomes, ferric iron (Fe^3+^) is reduced to ferrous iron (Fe^2+^) by STEAP3 and transported into the cytosol via DMT1. Fe^2+^ is subsequently exported across the abluminal membrane by ferroportin (FPN), supplying iron to brain interstitial fluid and neighboring astrocytes. After ischemic injury, local hepcidin expression increases and binds to FPN, inhibiting iron export and promoting endothelial iron retention, leading to brain iron dysregulation. The schematic illustrates the disruption of BBB iron trafficking in the context of ischemia-associated hepcidin upregulation.

Iron ranks as a highly abundant trace mineral in humans and is present in numerous organ systems such as the liver, spleen, kidneys, heart, skeletal muscle, and brain (4). About 65% of total body iron is found in hemoglobin, while the remaining 30 to 35% is stored primarily in the liver as ferritin. However, within the central nervous system, most iron crosses the blood–brain barrier (BBB) first by forming transferrin/transferrin receptor 1 (TFR/TFR1) endocytic complex between Fe^3+^ loaded transferrin and homodimeric TFR 1 on brain capillary endothelial cells (BCECs). Following complex formation, the cell membrane invaginates to form clathrin-coated vesicles, which are then internalized and mature into endosomes. Within the acidified endosome, Fe^3+^ detaches from transferrin and is converted to Fe^2+^ by ferroreductase enzymes (STEAP3), after which Fe^2+^ enters the cytosol via divalent metal transporter 1 (DMT1) (5). Once in the extracellular environment, iron can be absorbed by astrocytes or neurons, or can bind to transferrin and be transported into neurons through TfR-mediated uptake (6). Neurons, microglia, and astrocytes participate in the dynamic trafficking of iron, and astrocytes are believed to play a predominant role in controlling iron uptake in the brain (7). Myelin formation and maintenance in the central nervous system require substantial amounts of iron. Oligodendrocytes and their precursor cells constitute the cell population with the highest iron burden in the brain. Studies in rat brain sections have shown that mature oligodendrocytes contain the greatest levels of iron among glial cell types (8). Because iron is efficiently recycled and minimally excreted, systemic iron homeostasis largely relies on a balance between absorption and metabolic demand. The BBB regulates the entry of iron from the circulation into brain tissue, preventing direct exposure of central nervous system cells to circulating iron and other blood-derived substances and thereby maintaining a stable extracellular environment essential for neuronal function (9). Owing to the barrier function of the BBB, regulation of iron within the brain operates largely independently of whole-body iron balance, allowing cerebral iron stores to remain stable despite profound systemic iron depletion. In addition, the brain has a strong ability to store iron, and no efficient way of iron excretion from the brain tissue has been found, which means that the brain iron level is more likely to be overloaded and difficult to restore to normal levels (10). When this balance is disrupted, iron accumulation may occur, resulting in excessive iron deposition within the central nervous system. Such overload has been strongly linked to neurodegenerative disorders, including Alzheimer’s disease (AD) and Parkinson’s disease (PD) (11). Figure 1 depicts the mechanisms by which iron crosses the BBB under physiological conditions and highlights the disturbances that occur in response to ischemic insult.

## Disorders of iron metabolism affect PSCI

Brain cells acquire iron through three principal pathways: transferrin-bound iron endocytosis, non-transferrin-bound iron uptake, and ferritin-mediated transport. Under the regulation of hepcidin, Fe^2+^ entering BCECs is released into the interstitial fluid through ferrotransporter 1 (FPN1), part of which is oxidized to Fe^3+^ by ceruloplasmin on astrocytes and combined with transferrin to form transferrin bound iron, and the rest exists in the interstitial fluid in the form of free iron. Evidence from earlier research indicates that stroke impairs iron balance and triggers iron-induced neuronal damage, a process closely linked to poor prognosis following stroke (12). The occurrence of stroke will cause severe damage to the BBB, which causes the abnormal increase of the concentration of iron in the blood and penetrates into the brain tissue and deposits in the brain parenchyma. This phenomenon leads to an imbalance in brain tissue metabolism, which in turn leads to brain iron overload. The accumulation of Fe^2+^ may further aggravate neuronal damage and eventually lead to neuronal death. Excess intracellular Fe^2+^ participates in Fenton chemistry, generating highly reactive hydroxyl radicals that markedly amplify oxidative stress and lipid peroxidation. Nevertheless, such compensatory mechanisms can markedly increase the production of reactive oxygen species, thereby exacerbating neuronal damage and potentially causing neuronal degeneration or even cell death. As a result, abnormal iron metabolism eventually leads to neurological dysfunction, which seriously affects cognitive performance (13). Thus, serum iron levels appear to be partly associated with PSCI, and prior studies have reported that cognitive performance during the first 2 weeks after acute ischemic stroke (AIS) can predict substantial cognitive decline at 3–6 months (14). In an initial cross-sectional analysis of 313 ischemic stroke patients, researchers assessed whether serum iron concentrations were linked to cognitive deficits occurring in the early phase after acute ischemic stroke (15). In this study, participants were categorized into PSCI and non-PSCI groups. Serum iron concentrations were stratified into four levels, and binary logistic regression was performed to assess the relationship between serum iron and PSCI after adjusting for potential confounders. The study demonstrated that higher serum iron concentrations independently predicted a lower probability of cognitive impairment at 2 weeks post-ischemic stroke.

Importantly, the two principal mechanisms of post-stroke iron dysregulation, including hepcidin-driven intracellular iron retention and BBB disruption-mediated extracellular iron influx, likely affect different cellular compartments and collectively contribute to iron redistribution within the injured brain. Hepcidin upregulation following ischemia promotes FPN1 internalization in endothelial cells, astrocytes, and microglia, resulting in intracellular iron sequestration and expansion of the labile iron pool within these cells. In parallel, BBB breakdown permits the leakage of transferrin-bound and non-transferrin-bound iron into the extracellular space, increasing iron availability in the perivascular and interstitial compartments. Neurons may subsequently acquire excess iron through transferrin receptor-mediated uptake or other iron transport pathways, leading to intracellular accumulation particularly in metabolically vulnerable regions. Therefore, iron overload after stroke may not be restricted to a single compartment but rather reflects a dynamic redistribution process involving endothelial retention, glial sequestration, and neuronal uptake. This compartment-specific iron handling has important implications for therapeutic targeting, as strategies aimed at modulating hepcidin-FPN1 signaling may primarily affect intracellular iron retention, whereas approaches stabilizing BBB integrity may limit extracellular iron influx.

However, circulating serum iron levels do not necessarily reflect cerebral iron burden. The apparent protective association between higher serum iron levels and reduced early cognitive impairment should not be interpreted as evidence that systemic iron excess is beneficial. In the acute phase of stroke, reduced circulating iron levels are frequently driven by inflammation-induced functional iron deficiency rather than a true depletion of total body iron stores. Pro-inflammatory cytokines, particularly IL-6, upregulate hepatic expression of hepcidin (16). This leads to the internalization and degradation of ferroportin, resulting in iron sequestration within macrophages and hepatocytes, a hallmark of the anemia of inflammation. Under these conditions, low serum iron reflects an acute-phase redistribution rather than a decreased cerebral iron burden. Furthermore, functional iron deficiency may itself hamper neurological recovery by impairing mitochondrial respiration, reducing oxidative phosphorylation efficiency, and limiting the energy substrate required for synaptic repair and neuroplasticity (17). Recent clinical evidence further suggests that iron deficiency is independently associated with poorer neurological outcomes and an unfavorable prognosis after stroke (18). Collectively, these findings support a non-linear, U-shaped relationship between iron status and post-stroke outcomes, where both iron deficiency (impaired bioenergetics) and iron overload (ferroptosis-mediated toxicity) contribute to cognitive impairment through distinct pathophysiological mechanisms.

## Iron deposition and PSCI

Iron deposition represents a key feature of iron overload and has been strongly implicated in aging as well as in several neurodegenerative disorders (19). When iron excessively accumulates in the neurocyte, it disrupts mitochondrial redox activity and function, producing a reduction in membrane potential and ATP output that can culminate in neuronal degeneration and death (20, 21). Meanwhile, the BBB, which serves as a protective and metabolically regulating barrier for the brain, may likewise undergo disruption (22). Previous studies have demonstrated that acute iron overload exacerbates BBB disruption in hyperglycemic rat models, providing fundamental evidence for potential contributors to BBB injury in hyperglycemic patients with ischemic stroke (23). BBB damage commonly occurs during AIS, permitting excessive iron to accumulate across multiple brain areas, particularly the hippocampus, which is essential for memory integration. The resulting iron deposition triggers oxidative stress that promotes aberrant tau phosphorylation, neurofibrillary tangle formation, and subsequent disruption of neuronal synaptic plasticity (24), thus impairing brain memory and cognitive ability. This observation has been supported by measurements of iron content in hippocampal tissue sections from rat models (25). The basal ganglia is the main area of physiological iron deposition, which is responsible for motor coordination and cognitive regulation. Longitudinal studies on healthy middle-aged and elderly people have shown that the amount of iron deposition in the basal ganglia is directly proportional to the degree of brain atrophy, and also leads to the decline of cognitive function (26), related studies on the prefrontal cortex have also shown similar results (27). Therefore, the research on PSCI can be refined to different brain regions to evaluate the degree of cognitive impairment caused by different infarction sites, and may therefore contribute to predicting the prognosis of patients with acute stroke.

Hepcidin, a peptide hormone synthesized in the liver, controls iron balance by downregulating the activity of the iron exporter FPN1 (28). As a consequence, intestinal iron absorption decreases, leading to the retention of iron within macrophages and hepatocytes. Hepcidin regulates the release of intracellular iron by interacting with iron transporters on the cell membrane, and then affects the accumulation and distribution of iron. Previous studies have analyzed hepcidin levels in animal models of AIS and shown that its concentration is upregulated in the ischemic site of the brain (29). Similar to the results, follow-up studies showed that the serum hepcidin level of patients increased after the occurrence of ischemic stroke (30), which further verified the correlation between hepcidin and acute stroke. Brain ischemia can upregulate hepcidin expression via activation of the JAK/STAT3 signaling pathway, which promotes transferrin degradation and subsequent iron accumulation (31). Increased hepcidin levels suppress cellular iron export and enhance intracellular iron retention. These alterations correlate with the degree of cognitive impairment and the extent of structural brain changes (32). If validated in future prospective studies, quantitative assessment of hepcidin may hold promise as a diagnostic biomarker for PSCI.

Cerebral small vessel disease (CSVD) encompasses disorders affecting small arteries, arterioles, venules, capillaries, and their surrounding tissues. CSVD is strongly associated with stroke prognosis and the development of post-stroke cognitive impairment. Cerebral autosomal dominant arteriopathy with subcortical infarcts and leukoencephalopathy (CADASIL) represents a major subtype of CSVD and remains one of the most extensively investigated forms. Brain iron accumulation, particularly within deep gray matter structures, has been widely implicated in the pathophysiology of CADASIL. A susceptibility-weighted imaging (SWI) study further indicated that iron deposition in the putamen and caudate nuclei may correlate with clinical disease severity in CADASIL (33). Supporting this concept, a recent quantitative susceptibility mapping (QSM)-based study identified increased magnetic susceptibility in subcortical nuclei as a potential biomarker reflecting the severity of CADASIL (34). As CADASIL progresses, post-stroke cognitive impairment may develop, and the aforementioned findings indicate that nuclear iron deposition could serve as a novel marker for predicting PSCI severity. Recent evidence further shows that iron accumulation in the basal ganglia is associated with cognitive dysfunction in patients with CADASIL (35). White matter hyperintensities (WMH) are commonly detected on MRI in patients with CADASIL, and iron deposition has been linked to an increased WMH burden (36), on this basis, it is plausible that white matter microstructure acts as an intermediary pathway between iron deposition and PSCI in CADASIL. Supporting this notion, research has demonstrated that iron accumulation within the caudate nucleus and putamen contributes to microstructural white matter alterations in affected individuals (37). Jia et al. (34) employed peak width of skeletonized mean diffusivity (PSMD), a DTI-based metric, to assess white matter microstructural alterations and found that PSMD significantly mediated the association between brain iron deposition and cognitive performance. As iron deposition increased, PSMD values rose, accompanied by a decline in cognitive scores. However, current studies on the association between CSVD and cognitive impairment are relatively limited. Further longitudinal investigations are needed to establish the causal link between regional iron alterations and post-stroke cognitive impairment mediated by white matter microstructural damage in CSVD, as well as to assess how broadly these observations can be applied.

## Ferroptosis and PSCI

Ferroptosis, first described by Dixon et al. (38), is a regulated form of cell death driven by iron-dependent lipid peroxidation on cellular membranes. It is mechanistically distinct from apoptosis, necrosis, autophagy, and pyroptosis, and is characterized by excessive lipid peroxides, dysregulated intracellular iron metabolism, and impairment of antioxidant defense systems (39). Importantly, lipid peroxidation rather than generalized cytosolic or mitochondrial reactive oxygen species (ROS) generation is considered a defining trigger of ferroptosis. Accumulating experimental evidence suggests that ferroptosis-related mechanisms may contribute to neuronal injury after ischemic stroke and thereby participate in the pathogenesis of PSCI (40). Cerebral ischemia and ischemia–reperfusion injury disrupt brain iron homeostasis through blood–brain barrier dysfunction, inflammatory signaling, and altered expression of iron-regulatory proteins, leading to intracellular iron retention and enhanced lipid peroxidation (41). Under these conditions, iron-dependent lipid oxidative stress may promote ferroptosis-associated neuronal dysfunction, particularly within cognitive-relevant circuits such as the hippocampal and fronto-subcortical networks.

At the molecular level, impairment of the glutathione-glutathione peroxidase 4 (GSH-GPX4) axis is considered a central pathway involved in ferroptosis (42). GPX4 is the only known enzyme capable of reducing phospholipid hydroperoxides to non-toxic lipid alcohols, thereby preventing membrane damage (43). Experimental studies in ischemic stroke models have consistently demonstrated glutathione depletion and reduced GPX4 activity in affected brain regions, accompanied by increased lipid peroxidation and neuronal injury (16). Importantly, pharmacological inhibition of ferroptosis or restoration of GPX4 activity has been shown to attenuate neuronal damage and improve neurological outcomes in preclinical models (44), providing functional support for the involvement of ferroptosis-related pathways in ischemic brain injury. In addition to impaired antioxidant defenses, ischemia–reperfusion injury can activate ferritinophagy mediated by nuclear receptor coactivator 4 (NCOA4), leading to accelerated ferritin degradation and expansion of the intracellular labile iron pool (45). This process may further amplify iron-dependent lipid peroxidation and ferroptosis-associated neuronal injury. However, direct evidence linking ferritinophagy-driven ferroptosis to cognitive impairment remains limited (46), and further experimental and clinical studies are required to clarify its contribution to post-ischemic cognitive outcomes.

Notably, it should be emphasized that not all studies reporting iron accumulation or lipid peroxidation provide definitive evidence of ferroptosis. Reduced GPX4 activity, glutathione depletion, or increased lipid peroxidation are often interpreted as indirect indicators of ferroptosis, whereas rigorous confirmation requires additional molecular and functional criteria, including sensitivity to ferroptosis-specific inhibitors. Moreover, most evidence supporting a role for ferroptosis in PSCI is derived from animal or cellular models, and whether ferroptosis represents a dominant mechanism driving cognitive impairment in human stroke survivors remains to be fully established.

Collectively, available data support ferroptosis-related pathways as a plausible mechanistic link between post-stroke iron dysregulation, neuronal dysfunction, and subsequent cognitive decline within the spectrum of PSCI. Nevertheless, future longitudinal clinical studies integrating ferroptosis-related biomarkers, neuroimaging measures of brain iron deposition, and cognitive outcomes are required to clarify the causal contribution and translational relevance of ferroptosis in PSCI.

## Therapeutic strategies

The main prevention and treatment of PSCI is through the treatment of primary cerebrovascular diseases and the intervention of risk factors, and there is a lack of drug therapy to change the course of the disease. Consequently, there is a need to develop new therapeutic approaches based on underlying pathogenic mechanisms to improve patient outcomes. The following is a summary of the effects of iron chelator, diet therapy and TCM on iron metabolism disorders to improve the symptoms of PSCI. A summary of therapeutic strategies targeting iron dysregulation and ferroptosis in PSCI is provided in Table 1.

**Table 1:** Therapeutic strategies targeting iron metabolism dysregulation and ferroptosis in PSCI.

Therapeutic strategy	Interventions	Study model	Effects on iron metabolism	Outcomes	Proposed mechanisms	References
Iron chelation therapy	Deferoxamine (DFO)	MCAO mice; carotid artery ligation rats; aging and dementia mice	Chelation of excess iron; reduction of brain iron accumulation	Reduced infarct volume; improved learning and memory; reversal of post-stroke cognitive deficits	Activation of HIF-1α signaling; attenuation of oxidative stress and iron-mediated neurotoxicity	(40–44)
Dietary intervention	Ketogenic diet (KD)	AD mice; MCAO mice	Reduced cerebral iron deposition and lipid peroxidation	Improved cognitive performance; enhanced resistance to ischemia and hypoxia	Improved mitochondrial function; suppression of neuroinflammation and oxidative stress	(46–49)
Traditional Chinese medicine (TCM): monomer compounds	Gastrodin; QBT (ligustrazine-derived phthalide compound)	Vascular dementia mice	Regulation of iron homeostasis; reduced lipid peroxidation	Attenuated neuronal injury; improved cognitive function	Activation of Nrf2/Keap1/GPX4 and Nrf2/xCT/GPX4 pathways; inhibition of ferroptosis and inflammasome activation	(51, 52)
Traditional Chinese medicine (TCM): single herbs and extracts	Dendrobium extract	db/db mice	Suppression of iron-induced oxidative stress	Neuroprotection under ischemic and hypoxic conditions	Activation of Nrf2/GPX4 signaling pathway; inhibition of ferroptosis	(53)
Traditional Chinese medicine (TCM): compound formulas	Naotai Decoction; Bunao creams	MCAO rats; PSCI patients	Modulation of systemic and cerebral iron metabolism	Improved neurological recovery; increased MoCA and MMSE scores	Regulation of iron metabolism markers; antioxidant and neuroprotective effects	(54, 55)

### Treatment with iron chelators

Iron homeostasis within the brain is supported in part by the action of iron-chelating molecules. There are a variety of natural chelators in the human body, such as ascorbic acid and sugars, which can bind to iron ions in cells and tissues and participate in the process of iron metabolism and transport. Stroke can disrupt this metabolic balance, suggesting that exogenous iron chelators may represent a potential adjunctive therapeutic approach. Deferoxamine (DFO), developed over 50 years ago, is the most potent and widely used among the FDA-approved iron chelators (47). These agents were initially developed to treat systemic iron-overload conditions, such as transfusion-dependent thalassemia major, but their application has since expanded to neurological diseases. DFO has demonstrated substantial preclinical efficacy in stroke models, with intranasal administration (IN-DFO) significantly reducing infarct volume induced by middle cerebral artery occlusion (MCAO) in mice when administered either before or after the ischemic event (48), these findings are further corroborated by studies showing that systemic DFO administration is similarly effective in the rat models of carotid ligation-induced ischemia (49). In experimental models of neurodegenerative diseases, DFO has been proposed as a potential therapeutic agent for mitigating memory impairment associated with aging and dementia. DFO has been shown to attenuate age-related cognitive decline (50), and IN-DFO has been shown to enhance baseline memory task performance in young, healthy mice (51). With respect to VCI, DFO has been demonstrated to reverse cognitive deficits following both hemorrhagic and ischemic vascular insults in animal models (52), the underlying mechanism may involve DFO-induced activation of the HIF-1α pathway, which helps prevent ischemia-related cognitive impairment and confers neuroprotection following cerebrovascular occlusion (48).

Despite robust neuroprotective effects observed in experimental stroke models, clinical evidence supporting iron chelation therapy in stroke patients remains limited. Early-phase clinical studies evaluating deferoxamine have primarily focused on safety, feasibility, and biological target engagement, rather than cognitive outcomes, and have not demonstrated consistent functional or cognitive benefits. Moreover, uncertainties regarding optimal therapeutic timing, drug delivery to the brain, and the risk of inducing systemic or cerebral iron deficiency further complicate clinical translation. In addition, the clinical application of DFO is associated with potential risks, including acute iron toxicity, necessitating careful benefit–risk evaluation and strict adherence to therapeutic guidelines. To date, iron chelation therapy has not been recommended for the prevention or treatment of PSCI, highlighting the challenges of translating iron-mediated neurotoxicity from preclinical models into effective clinical interventions.

### Diet therapy

Ketogenic diet (KD) is a special dietary pattern characterized by high fat intake, very low carbohydrate content, and moderate protein (53). KD has demonstrated notable neuroprotective effects across various neurodegenerative disorders, including mild cognitive impairment (MCI), AD, PD, and amyotrophic lateral sclerosis. Its benefits span several mechanisms, such as enhancing mitochondrial function and biosynthetic capacity, suppressing inflammation and oxidative stress, modulating the gut microbiota, and strengthening cellular resistance to apoptosis (54). Recent studies have demonstrated that short-term KD reduces neuroinflammation, alleviates oxidative stress, and mitigates cognitive impairment in AD (55). In the AD mouse model, long-term and early KD induction reduced brain iron deposition and lipid peroxidation, thereby reducing neuronal cell damage caused by Aβ protein deposition and ferroptosis, thereby alleviating cognitive dysfunction in AD mice (56). Long-term adherence to KD has been reported to bolster neuronal resilience against ischemic and hypoxic injury, resulting in protective benefits during ischemic stroke (57).

At present, direct clinical evidence supporting ketogenic diet interventions in PSCI is lacking. However, recent small-scale clinical studies in patients with MCI and AD have reported modest cognitive benefits and metabolic improvements following KD. Whether such dietary strategies can modulate iron metabolism, ferroptosis-related pathways, or cognitive outcomes in PSCI populations remains to be determined in future targeted clinical trials.

### Mechanism-oriented multi-target therapeutic strategies

TCM is characterized by its ability to modulate cognition and vascular risk factors through diverse molecular targets and pathways (58). At present, TCM can be divided into single Chinese medicine compounds, single Chinese medicine and Chinese medicine compound. Studies have shown that certain TCM monomer compounds attenuate ferroptosis-related neuronal injury in experimental models of VCI through multiple mechanisms. These mechanisms include regulating iron ion balance, enhancing cellular antioxidant capacity, and reducing lipid peroxidation. For instance, gastrodin has been shown to suppress ferroptosis in hippocampal neurons by activating the Nrf2/Keap1-GPX4 signaling pathway, indicating that it may serve as an active therapeutic component for improving VCI through ferroptosis inhibition (59). QBT, a novel phthalic acid derivative extracted from Ligusticum chuanxiong, has been shown to mitigate neuronal injury and cognitive impairment in rats with vascular dementia by modulating the Nrf2/xCT/GPX4 and NLRP3/Caspase-1/GSDMD signaling pathways and inhibiting ferroptosis. These findings offer a new perspective and scientific rationale for developing future therapeutics targeting vascular dementia (60). Dendrobium extracts can prevent ferroptosis by activating Nrf2/GPX4 signaling pathway, thereby reducing oxidative stress response of cells under ischemic and hypoxic conditions, and exerting protective effects on the brain (61). Experimental evidence from a MCAO mouse model indicates that *Salvia miltiorrhiza* exerts neuroprotective effects by inhibiting ferroptosis, reducing lipid peroxidation, and preserving synaptic integrity, ultimately leading to improved post-stroke cognitive outcomes (62). It should be noted that the majority of evidence supporting the ferroptosis-modulating effects of traditional Chinese medicine is derived from cellular and animal models. Although these studies provide important mechanistic insights, their direct clinical relevance to PSCI remains to be established.

Naotai Decoction is a TCM formulation that has been used in clinical practice for the treatment of ischemic stroke. The drug can significantly reduce the damage of neurons in the MCAO rat model and promote faster recovery of nerve function (63). In a small single-center study evaluating Bunao cream in PSCI patients, the intervention group showed significant improvements in MOCA and MMSE scores compared with control group, along with reduced serum ferritin levels and increased serum iron, transferrin concentrations, and total iron-binding capacity relative to pretreatment values. These findings suggest that Bunao cream may enhance cognitive function, potentially through modulation of systemic iron metabolism. However, existing clinical studies of TCM interventions in PSCI are generally limited by small sample sizes, short follow-up durations, and heterogeneity in study design, and no therapeutic strategy specifically targeting iron dysregulation or ferroptosis has been recommended for routine clinical management of PSCI. Larger, well-designed randomized controlled trials are required to validate their efficacy and clarify the clinical relevance of ferroptosis- and iron-targeted mechanisms.

## Summary and prospect

PSCI represents one of the most prevalent subtype of VCI and imposes a substantial burden on patients’ functional independence and quality of life. Therefore, it is of great significance to early intervention and late treatment of PSCI. Based on the sorting of existing research results, this paper summarizes the related concepts of iron metabolism disorders, the related mechanisms that may lead to PSCI. Notably, systemic iron indices and brain iron deposition may not change in parallel after stroke. While elevated brain iron contributes to oxidative stress and neuronal injury, lower circulating serum iron levels in the acute phase may reflect inflammation-driven iron sequestration and could be associated with worse cognitive outcomes. These observations suggest a potentially non-linear or U-shaped relationship between iron status and post-stroke prognosis, highlighting the complexity of iron homeostasis in PSCI. Existing experimental evidence suggests that iron chelators, ketogenic dietary interventions, and certain traditional Chinese medicines may modulate neuronal iron homeostasis and ferroptosis-related pathways. However, their clinical efficacy in PSCI remains to be validated. At the same time, it also puts forward a new prospect: future research should focus on animal models of PSCI, further extend to the clinical population, and refine the mechanism and molecular research of related treatment methods.

## Glossary

AD: Alzheimer’s disease
AIS: Acute ischemic stroke
BBB: Blood–brain barrier
CNS: Central nervous system
CSVD: Cerebral small vessel disease
DFO: Deferoxamine
FPN1: Ferroportin 1
GSH: Glutathione
GPX4: Glutathione peroxidase 4
KD: Ketogenic diet
MCAO: Middle cerebral artery occlusion
MCI: Mild cognitive impairment
MoCA: Montreal Cognitive Assessment
MMSE: Mini-Mental State Examination
NCOA4: Nuclear receptor coactivator 4
Nrf2: Nuclear factor erythroid 2-related factor 2
PSCI: Post-stroke cognitive impairment
ROS: Reactive oxygen species
TCM: Traditional Chinese medicine
Tf: Transferrin
TfR1: Transferrin receptor 1
VCI: Vascular cognitive impairment
